# KIM-1 as a biomarker of kidney tubular damage in normoalbuminuric patients with type 2 diabetes mellitus and insulin resistance

**DOI:** 10.1590/2175-8239-JBN-2024-0123en

**Published:** 2025-05-19

**Authors:** Laura Eduarda de Oliveira, Clóvis Paniz, Rafael Noal Moresco, José Antonio Mainardi de Carvalho

**Affiliations:** 1 Universidade Federal de Santa Maria, Centro de Ciências da Saúde, Programa de Pós-Graduação em Ciências Farmacêuticas, Santa Maria, RS, Brazil.; 2 Universidade Federal de Santa Maria, Centro de Ciências da Saúde, Laboratório de Pesquisa em Análises Clínicas Aplicadas (LAPACA), Departamento de Análises Clínicas e Toxicológicas, Santa Maria, RS, Brazil.

**Keywords:** Diabetes, Mellitus, Kidney Disease, Insulin Resistance, Biomarkers

## Abstract

**Introduction:**

Insulin resistance (IR) is associated with diabetic kidney disease (DKD), and normoalbuminuric patients may present with renal tubular damage associated with insulin resistance (RTDAIR). In this context, the kidney injury molecule-1 (KIM-1) emerges as a useful biomarker for assessing tubular damage.

**Objective:**

To investigate the role of KIM-1 in the detection of RTDAIR in normoalbuminuric patients, as well as its relationship with HOMA-IR.

**Methodology:**

This was a cross-sectional study involving 82 normoalbuminuric patients with T2DM, divided into two groups according to HOMA-IR. Laboratory tests were performed, and data were analyzed using Student's t-test. A p-value < 0.05 was considered significant.

**Results:**

Patients with higher IR (HOMA-IR ≥ 2.77) had increased urinary KIM-1 (uKIM-1) concentrations (113.3 ± 65.0 ng/g creatinine), which may indicate incipient DKD.

**Conclusion:**

Increased HOMA-IR may be related to elevated uKIM-1 levels, suggesting that IR contributes to the development of RTDAIR. Furthermore, uKIM-1 may serve as a useful biomarker for its detection.

## Introduction

Insulin resistance is a condition that causes hyperglycemia and contributes to the development of type 2 diabetes mellitus (T2DM)^1^. These alterations could be detected using the Homeostasis Model Assessment of Insulin Resistance (HOMA-IR), which allows glycemic control and insulin resistance to be assessed^2^.

Patients with T2DM are more likely to develop diabetic kidney disease (DKD), diagnosed by albumin/creatinine ratio (ACR) levels. However, some studies have found the presence of renal tubular damage associated with changes in insulin resistance (RTDAIR) even in patients with ACR values within the normal range^3^. In this context, the kidney injury molecule (KIM-1) has been associated with changes in renal tubules, regardless of ACR levels^4^. Thus, the aim of this study was to investigate the role of KIM-1 in detecting tubular damage in T2DM patients and its relationship with the degree of insulin resistance, as assessed by HOMA-IR.

## Methods

This study included patients (n = 121) previously diagnosed with T2DM undergoing treatment at the outpatient clinic of the *Hospital Universitário de Santa Maria*, located in Santa Maria, Rio Grande do Sul, Brazil. The diagnosis of T2DM was established according to the American Diabetes Association (ADA) and the Brazilian Diabetes Society (SBD, in Portuguese) criteria, which consider blood glucose and glycated hemoglobin (HbA1c) values. Exclusion criteria included an ACR greater than 30 mg/g creatinine, urinary tract diseases, neoplasms, uncontrolled thyroid disorders, liver diseases, infections or inflammatory conditions, pregnancy, kidney transplantation, and the use of nephrotoxic drugs.

Of the initial 121 patients, 39 were excluded due to ACR concentrations exceeding 30 mg/g creatine. The remaining 82 patients were stratified into two groups based on HOMA-IR, following a study of a Brazilian population^5^: HOMA-IR ≤ 2.77 (n = 32) and HOMA-IR > 2.77 (n = 50). The presence of incipient DKD was determined using a uKIM-1 cut-off value (>109 ng/g creatinine), previously established for a Brazilian T2DM population, allowing discrimination between patients with and without incipient DKD^6^. This study was approved by the Ethics Committee for Human Studies of the Institution, under protocol number C.A.A.E 12303113.0.0000.5346, and all patients signed a consent form.

Blood samples were collected after overnight fasting using the venipuncture technique in Vacutainer® tubes (BD Diagnostics, Plymouth, UK). The samples were then centrifuged at 3,000 rpm for 15 minutes. In addition, all participants provided a urine sample, which was centrifuged at 1,500 rpm for 5 minutes. HbA1c concentrations were determined in whole blood samples by a chromatographic method with the D-10® analyzer (Bio-Rad, California, USA). Measurements of glucose, urinary creatinine and ACR were performed on the Dimension RxL Max® automated analyzer (Siemens Healthcare Diagnostics, Malvern, USA). uKIM-1 levels were analyzed using commercial sandwich ELISA kits (R&D systems, Minneapolis, MN, USA). Insulin levels were measured using the Cobas® 6000 analyzer (Roche Diagnostics, USA). HOMA-IR was calculated according to the equation: fasting insulin [μU/l] x fasting glucose [mmol/l]/22.5. The triglyceride-glucose index (TyG) was determined by the equation: ln [fasting triglycerides (mg/dl) x fasting glucose (mg/dl)]/2. The glomerular filtration rate (GFR) was estimated using the Chronic Kidney Disease Epidemiology Collaboration (CKD-EPI) creatinine equation.

Data were presented as mean and standard deviation (SD) for continuous variables. The distribution of variables was assessed using the Kolmogorov-Smirnov test. Student’s t-test was used to compare continuous variables, while the chi-square test was used for categorical variables. Statistical differences between groups were also assessed using Student’s t-test. A p-value < 0.05 was considered statistically significant. Statistical analyses were performed using GraphPad software (San Diego, CA, USA).

## Results

**Figure 1:**
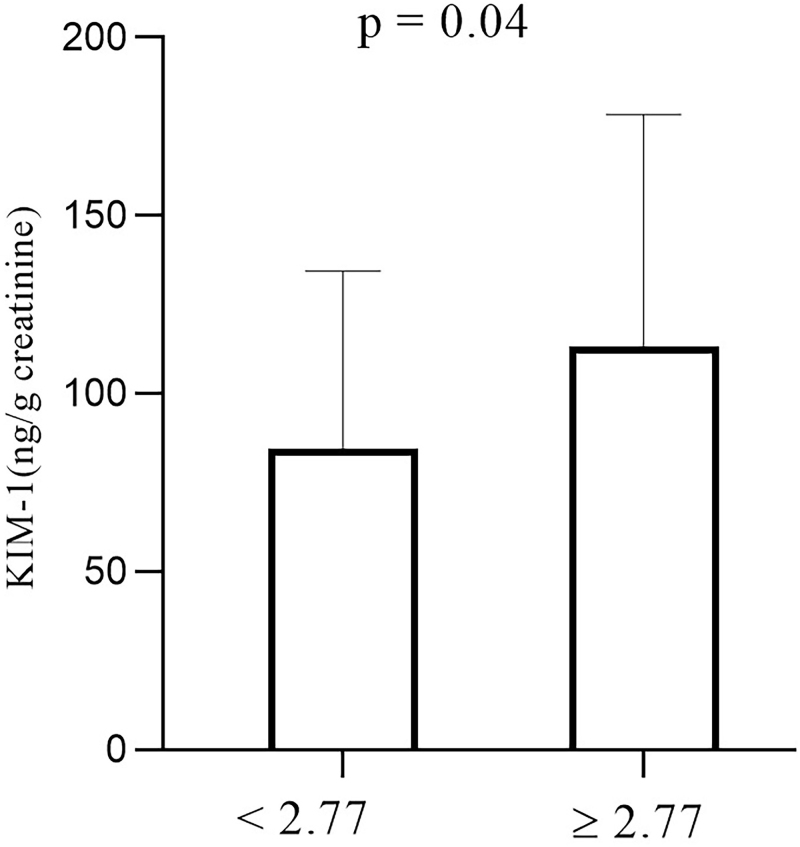
uKIM-1 values in T2DM patients with HOMA-IR < 2.77 and HOMA-IR ≥ 2.77. Data are expressed as mean ± standard deviation. Statistical differences between groups were assessed using Student’s t-test.

General data for the study population are described in Table 1. In the present study, patients in both groups had ACR values within the normal range, which, in theory, would indicate the absence of DKD. However, although ACR is one of the criteria adopted for diagnosing DKD, this condition may occur even with normal ACR results. The group with HOMA-IR ≥ 2.77 had a uKIM-1 value of 113.3 ± 65.0 ng/g creatinine, higher than the results found for the group with HOMA-IR < 2.77 which, in turn, presented a uKIM-1 concentration of 84.4 ± 50.0 ng/g creatinine (Figure 1). This difference represented an increase of approximately 35% in uKIM-1 results for the group with greater insulin resistance, which was statistically significant. In addition, patients with uKIM-1 >109 ng/g creatinine were observed in both groups, with a prevalence of 40% in the HOMA-IR ≥ 2.77 group and 15.6|% in the HOMA-IR < 2.77 group (p = 0.0193).

**Table 1:** Epidemiological and biochemical data of the study participants

	HOMA-IR index < 2.77	HOMA-IR index ≥ 2.77	*p*
Age (years)	63.7 ± 11.0	56.6 ± 13.9	0.018
BMI (kg/m^2^)	27.8 ± 4.3	33.2 ± 5.6	<0.001
Male Use of oral antihypertensives	13 (40.6%) 24 (75%)	15 (30.0%) 34 (68%)	0.322 0.496
Fasting glucose (mmol/L)	119.8 ± 30.7	138.4 ± 39.6	0.028
HbA1c (%)	6.7 ± 1.2	7.9 ± 2.0	0.003
Insulin (uUI/mL)	6.1 ± 2.1	21.5 ± 8.9	<0.001
Total cholesterol (mg/dL)	174.2 ± 33.6	170.8 ± 27.9	0.627
HDL cholesterol (mg/dL)	53.6 ± 12.0	44.6 ± 11.0	0.001
Triglycerides (mg/dL)	121.9 ± 56.7	140.4 ± 60.2	0.177
Creatinine (µmol/L)	1.1 ± 0.5	0.9 ± 0.4	0.095
TyG	4.7 ± 0.2	4.9 ± 0.3	0.008
HOMA-IR	1.7 ± 0.5	7.3 ± 3.7	<0.001
CKD-EPI (mL/min/1.73m^2^)	73.1 ± 19.8	76.02 ± 21.1	0.539
uCrea (mmol/mL)	0.9 ± 0.2	0.9 ± 0.2	0.477
ACR (mg/g creat)	7.5 ± 4.3	9.0 ± 6.3	0.242
uKIM-1(ng/g creat)	84.4 ± 50.0	113.3 ± 65.0	0.044

Abbreviations – BMI, body mass index; HbA1c, glycated hemoglobin; HDL, high-density cholesterol; TyG index, triglyceride-glucose index; HOMA-IR, Homeostasis Model Assessment of Insulin Resistance; CKD-EPI equation, creatinine equation from the Chronic Kidney Disease Epidemiology Collaboration; uCrea, urinary creatinine; ACR, albumin/creatinine ratio; uKIM-1, urinary kidney injury molecule-1. Notes – Continuous data are expressed as mean ± standard deviation. Categorical data are expressed as number of individuals and percentage (in parentheses).

## Discussion

The effects of hyperglycemia are known to be associated with various dysfunctions that could damage kidney structures and contribute to the development of DKD. The etiology of this condition is based on lesions caused by hyperfiltration of the glomerular filtration barrier, oxidative stress, and glomerular mesangial expansion, factors associated with poor glycemic control and the consequent increase in ACR concentrations^7^. It is therefore essential to assess the patient’s clinical condition using laboratory tests. In this context, uKIM-1 may be a relevant indicator, associated with insulin resistance biomarkers such as the HOMA-IR index, since KIM-1 could help detect tubular alterations before the concentration of ACR increases.

The gold standard for diagnosing DKD is the measurement of ACR, a biomarker of glomerular damage whose increase is the first sign of renal insufficiency^8^. However, studies have indicated the presence of histological alterations in the glomerular basement membrane in biopsy samples, even at ACR concentrations within the normal range^9,10^.

Considering that the effects of kidney injury caused by T2DM may lead to serious complications, it is crucial to screen the most susceptible patients or those in the early stages of the disease. Early diagnosis would enable the adoption of measures to prevent the development of DKD, and avoid serious and irreversible consequences^11^.

Therefore, efforts have been made to identify new biomarkers capable of detecting early kidney damage, including uKIM-1^12^. The use of this protein as a biomarker for RTDAIR offers several advantages, such as the ability to indicate the presence of tubulointerstitial injuries, since tubular injuries often precede glomerular damage. Furthermore, under normal conditions, uKIM-1 is undetectable in urine, which contributes to the increased specificity of this marker^13,14^.

The present study has some limitations, including the relatively low number of participants, the absence of sample size calculation, and the lack of data related to pharmacological and non-pharmacological treatments. However, we have demonstrated that individuals with greater insulin resistance may exhibit increased tubular damage, as evidenced by elevated uKIM-1 levels, even in the absence of glomerular damage (according to normal ACR values). As hyperglycemia is one of the main contributing factors to the development of renal tubular damage, individuals with greater insulin resistance tend to have poorer glycemic control^15^.

## Conclusion

In this study, we observed that patients with greater insulin resistance, indicated by HOMA-IR values, exhibited higher uKIM-1 concentrations, which may indicate the occurrence of kidney tubular damage among normoalbuminuric patients. uKIM-1, along with ACR, may be essential for identifying the presence of lesions in different nephron sites, such as the glomerulus and renal tubules, respectively. Therefore, the laboratory analysis of uKIM-1 has the potential to contribute to a more detailed and in-depth assessment of renal tubular alterations.

## Data Availability

The data for substantiating the findings of this manuscript are available with the corresponding author and can be made available on request.
