# Supplementary material for: KIM-1 as a biomarker of kidney tubular damage in normoalbuminuric patients with type 2 diabetes mellitus and insulin resistance
Source: J Bras Nefrol. 2025 May 19;47(3):e20240123. doi: 10.1590/2175-8239-JBN-2024-0123en (PMC12101797; doi:10.1590/2175-8239-JBN-2024-0123en)
Supplement: Translation

# Translation: KIM-1 como biomarcador de lesão tubular renal em pacientes com diabetes *mellitus* tipo 2 normoalbuminúricos e com resistência à insulina

**DOI:** 10.1590/2175-8239-JBN-2024-0123pt

**Authors:** Laura Eduarda de Oliveira, Clóvis Paniz, Rafael Noal Moresco, José Antonio Mainardi de Carvalho

**Affiliations:** 1 Universidade Federal de Santa Maria, Centro de Ciências da Saúde, Programa de Pós-Graduação em Ciências Farmacêuticas, Santa Maria, RS, Brasil.; 2 Universidade Federal de Santa Maria, Centro de Ciências da Saúde, Laboratório de Pesquisa em Análises Clínicas Aplicadas (LAPACA), Departamento de Análises Clínicas e Toxicológicas, Santa Maria, RS, Brasil.

**Keywords:** Diabetes, Mellitus, Nefropatias, Resistência à Insulina, Biomarcadores

## Abstract

**Introdução:**

A resistência à insulina (RI) está associada à doença renal diabética (DRD) e pacientes normoalbuminúricos podem apresentar lesão tubular renal associada à resistência à insulina (RTDAIR). Nesse contexto, a molécula de lesão renal-1 (KIM-1) se apresenta como um marcador útil para a avaliação da lesão tubular.

**Objetivo:**

Investigar o papel do KIM-1 na detecção de RTDAIR em pacientes normoalbuminúricos, bem como sua relação com o HOMA-IR.

**Metodologia:**

Estudo transversal envolvendo 82 pacientes com DM2 normoalbuminúricos, divididos em dois grupos pelo HOMA-IR. Foram realizados exames laboratoriais e os dados foram analisados utilizando o teste *t* de Student. Um valor de p < 0,05 foi considerado significativo.

**Resultados:**

Pacientes com maior RI (HOMA-IR ≥ 2,77) apresentaram concentrações de KIM-1 urinário (uKIM-1) mais elevadas (113,3 ± 65,0 ng/g creatinina), o que pode indicar DRD incipiente.

**Conclusão:**

O aumento do HOMA-IR pode estar relacionado à elevação do uKIM-1, sugerindo que a RI contribui para o desenvolvimento da RTDAIR. Além disso, o uKIM-1 pode ser um marcador útil para sua detecção.

## Introdução

A resistência à insulina é uma condição que causa hiperglicemia e contribui para o desenvolvimento do diabetes mellitus tipo 2 (DM2)^1^. Estas alterações podem ser detectadas por meio do Modelo de Avaliação da Homeostasia da Resistência à Insulina (HOMA-IR), que permite avaliar o controle glicêmico e a resistência à insulina^2^.

Pacientes com DM2 apresentam maior suscetibilidade ao desenvolvimento de doença renal diabética (DRD), diagnosticada pelos níveis da relação albumina/creatinina (RAC). Entretanto, alguns estudos constataram a presença de lesão tubular renal associada a alterações na resistência à insulina (RTDAIR) mesmo em pacientes com valores de RAC dentro da normalidade^3^. Nesse contexto, a molécula de lesão renal (KIM-1) tem sido associada a mudanças nos túbulos renais, independentemente do nível da RAC^4^. Assim, o objetivo deste estudo foi investigar o papel do KIM-1 na detecção da lesão tubular em pacientes com DM2 e sua relação com o grau de resistência à insulina, avaliado pelo HOMA-IR.

## Métodos

Este estudo incluiu pacientes (n = 121) previamente diagnosticados com DM2 em tratamento no ambulatório do Hospital Universitário de Santa Maria, localizado em Santa Maria, Rio Grande do Sul, Brasil. O diagnóstico de DM2 foi estabelecido conforme os critérios da *American Diabetes Association* (ADA) e da Sociedade Brasileira de Diabetes (SBD), que consideram os valores de glicemia e hemoglobina glicada (HbA1c). Os critérios de exclusão incluíram uma RAC superior a 30 mg/g de creatinina, doenças do trato urinário, neoplasias, doenças da tireoide não controladas, doenças hepáticas, infecções ou condições inflamatórias, gravidez, transplante renal e o uso de fármacos nefrotóxicos.

Dos 121 pacientes iniciais, 39 foram excluídos por apresentarem concentrações de RAC superiores a 30 mg/g creatina. Os 82 pacientes restantes foram estratificados em dois grupos com base no HOMA-IR seguindo um estudo de uma população brasileira^5^: HOMA-IR ≤ 2,77 (n = 32) e HOMA-IR > 2,77 (n = 50). A presença de DRD incipiente foi determinada considerando um ponto de corte para uKIM-1 (>109 ng/g creatinina), previamente estabelecido para uma população DM2 no Brasil, permitindo a discriminação entre pacientes com e sem DRD incipiente^6^. O presente estudo foi aprovado pelo Comite de Ética para Estudos em Seres Humanos da instituição, sob o número C.A.A.E 12303113.0.0000.5346, e todos os pacientes assinaram um formulário de consentimento.

As amostras de sangue foram coletadas após jejum noturno utilizando a técnica de punção venosa em tubos Vacutainer® (*BD Diagnostics*, Plymouth, Reino Unido). Em seguida, foram centrifugadas a 3.000 rpm durante 15 minutos. Além disso, todos os participantes forneceram uma amostra de urina, que foi submetida à centrifugação a 1.500 rpm por 5 minutos. A concentração de HbA1c foi determinada em amostras de sangue total por meio de um método cromatográfico, utilizando o analisador D-10® (*Bio-Rad*, Califórnia, EUA). As medições de glicose, creatinina urinária e RAC foram realizadas no analisador automático Dimension RxL Max® (*Siemens Healthcare Diagnostics*, Malvern, EUA). O uKIM-1 foi analisado por kits comerciais de ELISA em sanduíche (*R&D systems*, Minneapolis, MN, EUA). A insulina foi medida com o analisador Cobas® 6000 (*Roche Diagnostics*, EUA). O HOMA-IR foi calculado pela equação: insulina em jejum [μU/l] x glucose em jejum [mmol/l]/22,5. O índice triglicérides-glicose (TyG) foi determinado pela equação: ln [triglicérides em jejum (mg/dl) x glicose em jejum (mg/dl)]/2. A taxa de filtração glomerular (TFGe) foi estimada utilizando a equação de creatinina da *Chronic Kidney Disease Epidemiology Collaboration* (CKD-EPI).

Os dados foram apresentados como média e desvio padrão (DP) para variáveis contínuas. A distribuição das variáveis foi testada por meio do teste de Kolmogorov-Smirnov. O teste *t* de Student foi empregado para comparar variáveis contínuas, enquanto o teste qui-quadrado foi utilizado para variáveis categóricas. As diferenças estatísticas entre os grupos também foram avaliadas pelo teste *t* de Student. Um valor de p < 0,05 foi considerado estatisticamente significativo. As análises estatísticas foram realizadas com o software GraphPad (San Diego, CA, EUA).

## Resultados

Os dados gerais da população do estudo estão descritos na Tabela 1. Neste estudo, os pacientes de ambos os grupos apresentaram valores de RAC dentro da faixa de normalidade, o que, em teoria, indicaria a ausência de DRD. No entanto, embora a RAC seja um dos critérios adotados para o diagnóstico de DRD, esta patologia pode ocorrer mesmo com resultados normais de RAC. O grupo com HOMA-IR ≥ 2,77 apresentou um valor de uKIM-1 de 113,3 ± 65,0 ng/g creatinina, superior aos resultados encontrados para o grupo com HOMA-IR < 2,77 que, por sua vez, apresentou uma concentração de uKIM-1 de 84,4 ± 50,0 ng/g creatinina (Figura 1). Essa diferença representou um incremento de aproximadamente 35% nos resultados de uKIM-1 no grupo com maior resistência insulínica, sendo estatisticamente significativa. Além disso, pacientes com uKIM-1 >109 ng/g creatinina foram observados em ambos os grupos, com uma prevalência de 40% no grupo HOMA-IR ≥ 2,77 e 15,6|% no grupo HOMA-IR < 2,77 (p = 0,0193).

**Tabela 1:** Dados epidemiológicos e bioquímicos dos participantes do estudo

	Índice HOMA-IR < 2,77	Índice HOMA-IR ≥ 2,77	*p*
Idade (anos)	63,7 ± 11,0	56,6 ± 13,9	0,018
IMC (kg/m^2^)	27,8 ± 4,3	33,2 ± 5,6	<0,001
Homens Uso de anti-hipertensivo oral	13 (40,6%) 24 (75%)	15 (30,0%) 34 (68%)	0,322 0,496
Glicose de jejum (mmol/L)	119,8 ± 30,7	138,4 ± 39,6	0,028
HbA1c (%)	6,7 ± 1,2	7,9 ± 2,0	0,003
Insulina (uUI/mL)	6,1 ± 2,1	21,5 ± 8,9	<0,001
Colesterol total (mg/dL)	174,2 ± 33,6	170,8 ± 27,9	0,627
Colesterol HDL (mg/dL)	53,6 ± 12,0	44,6 ± 11,0	0,001
Triglicerídeos (mg/dL)	121,9 ± 56,7	140,4 ± 60,2	0,177
Creatinina (µmol/L)	1,1 ± 0,5	0,9 ± 0,4	0,095
TyG	4,7 ± 0,2	4,9 ± 0,3	0,008
HOMA-IR	1,7 ± 0,5	7,3 ± 3,7	<0,001
CKD-EPI (mL/min/1,73m^2^)	73,1 ± 19,8	76,02 ± 21,1	0,539
uCrea (mmol/mL)	0,9 ± 0,2	0,9 ± 0,2	0,477
RAC (mg/g creat)	7,5 ± 4,3	9,0 ± 6,3	0,242
uKIM-1(ng/g creat)	84,4 ± 50,0	113,3 ± 65,0	0,044

Abreviações – IMC, índice de massa corporal; HbA1c, hemoglobina glicada; HDL, colesterol de alta densidade; índice TyG, índice triglicérides-glicose; HOMA-IR, Modelo de Homeostase da Resistência à Insulina; taxa CFD-EPI, equação de creatinina da *Chronic Kidney Disease Epidemiology Collaboration*; uCrea, creatinina urinária; RAC, relação albumina/creatinina; uKIM-1, molécula-1 de lesão renal urinária. Notas – Os dados contínuos são expressos em média ± desvio padrão. Os dados categóricos são expressos em número de indivíduos e porcentagem (entre parênteses).

**Figura 1:** Valores de uKIM-1 em pacientes com DM2 com índice HOMA-IR < 2,77 e HOMA-IR ≥ 2,77. Os dados são expressos como média ± desvio padrão. As diferenças estatísticas entre os grupos foram avaliadas usando o teste *t* de Student.

## Discussão

Sabe-se que os efeitos da hiperglicemia estão associados a diversas disfunções que podem causar danos às estruturas renais e contribuir para o desenvolvimento de DRD. A etiologia dessa condição baseia-se em lesões causadas pela hiperfiltração da barreira de filtração glomerular, estresse oxidativo e expansão mesangial glomerular, fatores associados ao controle glicêmico inadequado e ao consequente aumento da concentração da RAC^7^. Dessa forma, a avaliação do estado clínico do paciente por meio de exames laboratoriais é fundamental. Nesse contexto, o uKIM-1 pode ser um indicador relevante, associado a marcadores de resistência à insulina, como o índice HOMA-IR, uma vez que o KIM-1 pode contribuir para a detecção de alterações tubulares antes do aumento da concentração da RAC.

O padrão ouro para o diagnóstico da DRD é a dosagem da RAC, um marcador de dano glomerular cujo aumento constitui o primeiro sinal de insuficiência renal^8^. Entretanto, estudos têm indicado a presença de alterações histológicas na membrana basal glomerular em amostras de biópsia mesmo em concentrações de RAC dentro da faixa de normalidade^9,10^.

Considerando que os efeitos da lesão renal causada pelo DM2 podem levar a complicações graves, é importante fazer a triagem dos pacientes mais suscetíveis ou daqueles que se encontram nas fases iniciais da doença. O diagnóstico precoce permitiria a adoção de medidas para prevenir o desenvolvimento da DRD e evitar consequências graves e irreversíveis^11^.

Por conseguinte, tem-se buscado identificar novos marcadores capazes de detectar lesões renais precoces, incluindo o uKIM-1^12^. A utilização desta proteína como marcador de RTDAIR oferece muitas vantagens, como a capacidade de indicar a presença de lesões tubulointersticiais, uma vez que as lesões tubulares frequentemente precedem as lesões glomerulares. Além disso, em condições normais, o uKIM-1 é indetectável na urina, o que contribui para o aumento da especificidade deste marcador^13,14^.

O presente estudo apresenta algumas limitações, incluindo o número relativamente baixo de participantes, a ausência de cálculo do tamanho amostral e a falta de alguns dados relacionados aos tratamentos farmacológico e não farmacológico. No entanto, demonstramos que indivíduos com maior resistência à insulina podem apresentar dano tubular aumentado, conforme evidenciado pelo aumento de uKIM-1, mesmo na ausência de dano glomerular (de acordo com os valores normais de RAC). Como a hiperglicemia é um dos principais fatores contribuintes para o desenvolvimento de lesão tubular renal, indivíduos com maior resistência à insulina tendem a apresentar um pior controle glicêmico^15^.

## Conclusão

Neste estudo, observamos que pacientes com maior resistência à insulina, indicada pelos valores de HOMA-IR, apresentaram concentrações mais elevadas de uKIM-1, o que pode indicar a ocorrência de lesão tubular renal dentre os pacientes normoalbuminúricos. O uKIM-1, juntamente com a RAC, pode ser fundamental para identificar a presença de lesões em diferentes locais do néfron, como o glomérulo e os túbulos renais, respectivamente. Portanto, a análise laboratorial do uKIM-1 apresenta potencial de contribuir para uma avaliação mais detalhada e aprofundada das alterações ocorridas a nível tubular renal.

## Data Availability

Os dados que fundamentam os achados deste manu-scrito estão disponíveis com o autor correspondente e podem ser disponibilizados mediante solicitação.
